# Longitudinal Outcomes of the COVID-19 Pandemic on Youth Physical Fitness

**DOI:** 10.1001/jamanetworkopen.2025.13721

**Published:** 2025-06-04

**Authors:** Andjelka Pavlovic, David Leonard, Timothy J. Walker, Derek W. Craig, Jizya Injil, Laura F. DeFina

**Affiliations:** 1 Kenneth H. Cooper Institute, Texas Tech University Health Sciences Center, Dallas; 2 UTHealth Houston School of Public Health, Houston, Texas

## Abstract

**Importance:**

The COVID-19 pandemic led to a decline in physical activity levels among youths, which could have a negative impact on their fitness.

**Objective:**

To examine longitudinal changes in cardiorespiratory fitness (CRF) and musculoskeletal fitness (MSF) among students at schools participating in a school-based physical activity program during fall 2019 through spring 2023.

**Design, Setting, and Participants:**

This longitudinal cohort study included US schools reporting CRF and MSF data at least once during the pandemic (fall 2020 to fall 2021) and at least 1 additional time before or after. In a subset of schools for which learning modality data was available, the association between in-person vs remote or hybrid environments and fitness was examined.

**Exposures:**

COVID-19 pandemic status and school learning modality.

**Main Outcomes and Measures:**

CRF and MSF results were entered into the fitness assessment software by school staff. Estimated maximal oxygen consumption (milliliters per kilogram per minute) was categorized into 1 of 3 groups (healthy fitness zone [HFZ], needs improvement, and health risk), whereas MSF was categorized into 2 groups (HFZ or needs improvement) according to age-specific and sex-specific criterion-referenced standards. Mixed-effects logistic regression was used to estimate adjusted HFZ odds ratios (ORs) for CRF (vs needs improvement and health risk) and MSF (vs needs improvement).

**Results:**

The sample consisted of 264 schools and 152 094 students (77 818 boys [51.2%]) across 21 states. Compared with the prepandemic and postpandemic periods, students were significantly less likely to achieve the CRF HFZ (OR, 0.72; 95% CI, 0.66-0.78) and MSF HFZ (OR, 0.82; 95% CI, 0.75-0.89) during the pandemic. Adjusted CRF was 0.55 mL/kg/minute lower in girls (95% CI, 0.36-0.74 mL/kg/minute) and 0.86 mL/kg/minute (95% CI, 0.63-1.10 mL/kg/minute) lower in boys during the pandemic (*P* < .001). Among students at 116 schools with available learning modality by week, those in remote or hybrid environments for 15 to 22 weeks were significantly more likely to achieve the CRF HFZ compared with those who were hybrid or remote for shorter periods of time (15-22 vs 0-4 weeks, OR, 1.26; 95% CI, 1.06-1.50; 15-22 vs 5-14 weeks, OR, 1.34; 95% CI, 1.10-1.63).

**Conclusions and Relevance:**

In this cohort study of schools, a COVID-19–related decline in youth physical fitness was observed. Compared with prepandemic and postpandemic periods, CRF and MSF HFZ achievement were significantly lower during the pandemic, but the reduction did not appear to be associated with extended remote or hybrid environments.

## Key Points

**Question:**

What were the longitudinal outcomes of the COVID-19 pandemic on youth physical fitness, and how were learning modalities associated with these changes?

**Findings:**

This cohort study included 264 schools and 152 094 students across the US. Compared with the prepandemic and postpandemic periods, students were significantly less likely to achieve the healthy fitness zone for cardiorespiratory and musculoskeletal fitness during the pandemic, but the reduction was not associated with extended remote or hybrid environments.

**Meaning:**

Should other catastrophic world events occur in the future, health care practitioners, teachers, and parents should have a plan in place aimed at maintaining the physical fitness of youths.

## Teaser

This cohort study examines whether the COVID-19 pandemic was associated with declines in cardiorespiratory and musculoskeletal fitness among US youths.

## Introduction

Beginning in December 2019, the previously unknown pneumonia-like virus, SARS-CoV-2, began to appear in China. The number of medical cases of COVID-19, the illness caused by SARS-CoV-2, grew at an unprecedented rate and was declared a pandemic by the World Health Organization in March 2020. In response to the pandemic, most countries around the world implemented various societal measures for the purpose of containing the virus. For youths specifically, these measures included social distancing, school closures, sheltering in place, mandatory face masks in public spaces, and cancellation of such activities as sports, large events, and group gatherings.^1^

Approximately 3 years later, the US Department of Health and Human services declared the end of the federal COVID-19 public health emergency. Throughout the 3-year period, youths around the nation were subjected to major disturbances in their daily routines that, in many instances, led to negative changes in their physical and mental health. To date, studies have shown that the COVID-19–related school closures resulted in substantial learning losses for youths enrolled in grades kindergarten through 12,^2^ in fact, concluding that many students made little to no progress while learning from home. In addition to the observed declines in learning, Hawes et al^3^ reported that children were experiencing mental health issues, such as anxiety and depression, and this was especially true for female adolescents.

The negative consequences of the COVID-19 pandemic were not limited to academic performance and mental health. Studies^4,5^ have also reported declines in physical health of youths around the world. For example, Lange et al^4^ observed substantial increases in body mass index (BMI) among a large cohort of 432 302 children aged 2 to 19 years living in California, with youths who had overweight or obesity presenting with the largest gains in BMI. These findings are speculated to be due to school closures, disturbances in daily routines, stress, and decreases in physical activity throughout the pandemic.

During the early stages of the pandemic, studies found a substantial decline in physical activity among youths in China,^6^ Italy,^7^ and the US.^8^ For example, in China, the median time spent in physical activity decreased from 540 minutes per week to 105 minutes per week. This finding was coupled with an increase in prevalence of inactive youths (21.3% prepandemic vs 65.6% during early stages of the pandemic).^6^ In Italy, children and adolescents decreased time spent in sports by 2.3 hours per week and increased screen time by approximately 5 hours per week.^7^ These trends continued well into the pandemic, where a systematic review of literature found that physical activity of youths declined greatly during the first 1.5 years of the pandemic.^9^ However, most of the aforementioned studies did not directly assess the 2 primary health-related physical fitness components in youths (cardiorespiratory endurance and muscular strength) using objective and validated testing methods.

Regular participation in moderate-to-vigorous intensity physical activity improves health-related physical fitness, specifically cardiorespiratory fitness (CRF) and musculoskeletal fitness (MSF), which is associated with well-established health benefits and decreased likelihood of severe complications from the COVID-19 virus.^10^ To date and to our knowledge, no study has examined the longitudinal trends of physical fitness measured objectively before, during, and after the COVID-19 pandemic in schools across the US. Therefore, the purpose of this study was 2-fold: (1) examine schoolwide CRF and MSF healthy fitness zone (HFZ) achievement throughout the COVID-19 pandemic (fall 2019 to spring 2023), and (2) examine the association between weeks spent in remote or hybrid learning and CRF and MSF HFZ achievement among a large cohort of schools participating in the National Football League (NFL) PLAY 60 FitnessGram Project.

## Methods

This cohort study adhered to the Strengthening the Reporting of Observational Studies in Epidemiology (STROBE) reporting guidelines for epidemiological research. School-level demographic information, including sex, race and ethnicity, socioeconomic status, and school setting, was based on the categories from the National Center for Education Statistics (NCES) database. Data on race and ethnicity are included in the study to show that the diversity of the sample is similar to that of US schools. Health-related physical fitness data were collected as part of standardized physical education (PE) practices.

### School-Based Physical Activity Program

This study consisted of schools enrolled in the NFL PLAY 60 FitnessGram Project, a collective impact initiative of The Cooper Institute and funded by the NFL Foundation. The program is implemented in all NFL markets and their surrounding areas, and the participating schools and districts are chosen on the basis of the following criteria: (1) NFL club priorities, (2) geographic location of the district, (3) qualification for federal financial assistance (ie, Title I eligibility), and (4) size of district to allow for project expansion over time. Once enrolled, the program provides schools with incentives (eg, NFL Flag kits, fitness equipment, and FitnessGram kits) and resources (eg, PLAY 60 App, EVERFI learning modules, and physical activity promotion materials) aimed at improving the health and wellness of youths. A PE teacher at each school is responsible for implementing the project and completing the programmatic requirements. Each semester, the PE teacher administers a health-related physical fitness assessment and inputs the results into the FitnessGram Software. The final sample of schools reported CRF and MSF data at least once during the pandemic (fall 2020 to fall 2021) and at least 1 additional time before (fall 2019 to spring 2020) or after (spring 2022 to spring 2023). School-level agreements were obtained for all participating schools. Subsequently, only deidentified data were provided to the researchers. The project is reviewed and approved annually by the institutional review board of The Cooper Institute. Because no data for individual students were collected, the study did not require informed consent, in accordance with 45 CFR §46.

### Fitness Assessment

The health-related fitness assessment battery was created by The Cooper Institute in 1982 to provide PE teachers with a tool to assess and track youth physical fitness as it relates to health-related criterion. Today, the assessment is one of the most widely used physical fitness assessments, both nationally and internationally. The assessment evaluates the traditional health-related components of fitness, including CRF, MSF, flexibility, and body composition. Scores obtained from the assessment are evaluated against objective sex-specific and age-specific criterion-referenced standards, referred to as healthy fitness zone (HFZ) standards. Some common assessments used include the following: PACER (progressive aerobic cardiovascular endurance run) test, 1-mile run or walk, push-up, curl-up, and BMI. For the purpose of the present study, the variables of interest were CRF (PACER test 1-mile walk or run) and MSF (push-up test). In an effort to enhance data quality and reduce measurement error, standardized assessment and software training were provided to participating schools. All data obtained from the physical fitness assessments were logged into a cloud-based data entry and tracking platform (GreenLight Fitness, LLC).

### Cardiorespiratory Fitness

CRF was estimated from the PACER, 1-mile run, or 1-mile walk tests. The PACER test, commonly known as the *beep test*, requires students to run back and forth over a specific distance (ie, either 15 or 20 m) at a pace indicated by a beep. The assessment starts at a slow jogging pace and gradually increases in speed with each stage change. The students are instructed to run until they can no longer sustain the pace of the cadence. Upon conclusion of the chosen CRF assessment, each student’s recorded number of completed laps, or time to completion for the 1-mile run or walk assessments, is logged into the software and the number per time is converted into an estimated maximal oxygen consumption (milliliters per kilogram per minute) or CRF level. The results are then further categorized according to age-specific and sex-specific standards into 1 of 3 groups: HFZ, needs improvement (NI), and needs improvement–health risk (NI-HR). The criterion-referenced categories are scientifically linked to health outcomes in youths, such that if a student achieves the NI-HR category based on PACER performance, he or she is at an increased risk for metabolic syndrome. Additional information regarding CRF protocols has been described elsewhere.^11^

### Musculoskeletal Fitness

MSF was measured with a 90-degree push-up test performed at a cadence of approximately 1 push-up every 3 seconds or 20 push-ups per minute. To begin the assessment, each student assumes a prone position with hands under or slightly wider than the shoulders. The performer pushes off the mat by extending their arms with the back in a straight line from head to toe throughout the test. Each student is instructed to lower their body until the elbows attain a 90-degree angle. The movement is repeated as many times as possible, without breaking form, according to the established cadence referenced above. The resulting score is then categorized into either the HFZ or the NI zone. Additional information regarding the push-up protocols has been published elsewhere.^11^

### School Learning Modality During the COVID-19 Pandemic

School characteristics (eg, campus type, setting, Title I eligibility, and demographics) were based on data from the NCES, a publicly available database that is part of the US Department of Education. School learning modality status by week during August 2020 to December 2022 from the US Centers for Disease Control and Prevention was linked to a subset of schools by NCES school identification. The schools were grouped into 3 categories of hybrid or remote learning according to the distribution of the data: 0 to 4 weeks, 5 to 14 weeks, and 15 to 22 weeks.

### Statistical Analysis

School characteristics including enrollment and demographics were linked to schools participating in the school-based physical activity program by NCES school identification. Assessment measurements on individual students were clustered by school. Mixed-effects linear regression was used to estimate CRF (milliliters of oxygen per kilogram per minute) during the pandemic (fall 2020 to fall 2021) compared with before or after (fall 2019 to spring 2020, spring 2022 to spring 2023). Sex (female vs male), grade (7-12 vs 1-6), Title I status (ineligible vs eligible or unknown), and US Census region (Midwest, South, or West vs Northeast) were entered along with time period as fixed effects. Random effects between schools and within schools across time periods were included to accommodate correlation of repeated measurements. Mixed-effects logistic regression was similarly used to estimate adjusted HFZ odds ratios (ORs) for CRF (vs NI and NI-HR) and MSF (vs NI). Learning modality (in-person, remote, or hybrid) by week during fall 2020 to fall 2021 was available in a subgroup of schools and linked by NCES school identification. Mixed-effects logistic regression was used to estimate adjusted HFZ ORs for CRF and MSF in schools with 0 to 4, or 5 to 14 vs 15 to 22 weeks of hybrid or remote learning adjusted for sex, grade, Title I status, and region. Mixed-effects regression does not require balanced data and provides unbiased inference in the case of dependent data missing at random.^12^ The level of statistical significance was set at 2-sided *P* < .05, and all analyses were programmed in SAS/STAT statistical software version 9.4 (SAS Institute).

## Results

The sample consisted of 264 schools (197 elementary schools [74.6%], 42 junior or middle schools [15.9%], 23 high schools [8.7%], and 2 other schools [0.8%]) and 152 094 students (77 818 boys [51.2%]) across 63 districts in 21 states (Table 1). Most participating schools were located in urban areas (116 schools [43.9%]) and had Title I eligibility (196 schools [74.2%]). Additionally, each region of the US (Northeast, Midwest, South, and West) had a minimum of 42 schools. The number of schools that assessed CRF and MSF and input data into the software varied throughout the period of this study (Table 2). For example, due to restrictions imposed by the COVID-19 pandemic and the recruitment structure of the program, the number of schools reporting fitness data ranged from 45 in fall 2020 to 237 in fall 2021, whereas the number of students assessed ranged from 6920 in fall 2020 to 62 235 in spring 2023.

**Table 1.** Characteristics of Schools Reporting Cardiorespiratory and Musculoskeletal Outcomes Before, During, and After the COVID-19 Pandemic (Fall 2019 to Spring 2023)^a^

Characteristics	Schools, No. (%) (N = 264)
No. of school districts	63
Type of schools	
Elementary	197 (74.6)
Middle	42 (15.9)
High	23 (8.7)
Other	2 (0.8)
School setting^b^	
Urban	116 (43.9)
Suburban	89 (33.7)
Town	25 (9.5)
Rural	34 (12.9)
Region	
Northeast	43 (16.3)
Midwest	65 (24.6)
South	114 (43.2)
West	42 (15.9)
Title I eligible (low socioeconomic status schools)^b^^,^^c^	196 (74.2)
No. of students in participating schools	152 105
Student sex^b^	
Female	74 276 (48.8)
Male	77 818 (51.2)
Unknown	11 (<0.1)
Student race and ethnicity^b^	
African American or Black	35 279 (23.2)
American Indian or Alaska Native	716 (0.5)
Asian or Pacific Islander	9746 (6.4)
Hawaiian or Pacific Islander	214 (0.1)
Hispanic	52 335 (34.4)
White	46 729 (30.7)
≥2 Races	7075 (4.7)
Unknown	11 (<0.1)
No. of students assessed per semester	
Fall 2019	43 792
Spring 2020	22 160
Fall 2020	6920
Spring 2021	18 111
Fall 2021	45 197
Spring 2022	54 739
Fall 2022	54 793
Spring 2023	62 235

^a^ A total of 261 schools reported cardiorespiratory fitness, and 259 reported musculoskeletal fitness.

^b^ Data are based on categories provided by the National Center for Education Statistics for 2022 to 2023 school year.

^c^ Seventeen schools had missing data on Title I eligibility.

**Table 2.** Schools Reporting Fitness Outcomes Data Before, During, and After the COVID-19 Pandemic (Fall 2019 to Spring 2023)^a^

Variable	Mean (SD)							
	Fall 2019 (n = 137)	Spring 2020 (n = 69)	Fall 2020 (n = 45)	Spring 2021 (n = 85)	Fall 2021 (n = 237)	Spring 2022 (n = 228)	Fall 2022 (n = 184)	Spring 2023 (n = 182)
Cardiorespiratory fitness								
No. of schools	133	58	38	69	216	207	173	169
Maximal oxygen consumption, mL/kg/min	42.3 (3.2)	43.2 (3.2)	41.3 (2.5)	41.3 (3.0)	40.8 (3.3)	41.4 (3.7)	41.3 (3.7)	41.9 (3.6)
Healthy fitness zone, %	56.9 (21.8)	62.2 (17.6)	49.3 (23.4)	48.6 (23.1)	43.4 (27.3)	48.1 (26.7)	45.6 (24.3)	50.8 (22.8)
Needs improvement, %	25.1 (13.4)	21.8 (11.9)	30.2 (16.4)	26.9 (12.9)	29.1 (16.1)	25.1 (13.6)	29.9 (16.0)	26.8 (13.0)
Needs improvement–health risk, %	18.0 (18.4)	16.0 (11.0)	20.6 (18.7)	24.5 (19.8)	27.5 (24.4)	26.8 (23.5)	24.4 (22.1)	22.4 (18.3)
Muscular strength and endurance								
No. of schools	127	53	34	72	212	215	172	169
No. of completed push-ups	9.6 (4.7)	10.9 (3.6)	9.5 (4.7)	10.7 (4.2)	8.4 (3.7)	9.7 (4.2)	9.0 (4.7)	9.7 (3.9)
Healthy fitness zone, %	51.0 (20.8)	59.0 (19.5)	52.1 (23.6)	57.3 (19.8)	48.3 (21.0)	53.7 (20.6)	49.7 (21.5)	53.8 (19.8)
Needs improvement, %	49.0 (20.8)	41.0 (19.5)	47.9 (23.6)	42.7 (19.8)	51.7 (21.0)	46.3 (20.6)	50.3 (21.5)	46.2 (19.8)

^a^ For each semester, estimated cardiorespiratory and musculoskeletal fitness outcomes and their respective age-specific and sex-specific healthy fitness zone achievement are presented.

**Figure 1.**
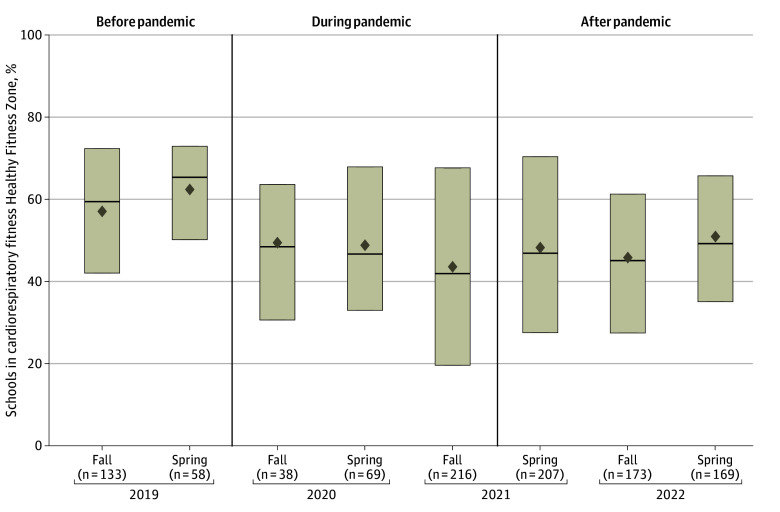
Unadjusted Cardiorespiratory Fitness Healthy Fitness Zone Achievement by Semester With Number of Schools Reporting Boxes extend from the 25th to the 75th percentiles, medians are indicated by horizontal bars, and means are indicated by diamonds.

The unadjusted CRF HFZ achievement for each semester is shown in Figure 1. After adjusting for grade level, sex, Title I eligibility, and region, students were significantly less likely to achieve HFZ for CRF during the pandemic (OR, 0.72; 95% CI, 0.66-0.78) compared with the prepandemic and postpandemic periods (Table 3). There was a significant decrease in CRF in youths during the COVID-19 pandemic for both boys and girls. Adjusted CRF was 0.55 mL/kg/minute (95% CI, 0.36-0.74 mL/kg/minute) lower in girls and 0.86 mL/kg/minute (95% CI, 0.63-1.10 mL/kg/minute) lower in boys during the pandemic (*P* < .001). Similarly, in the fully adjusted model, HFZ achievement for MSF was significantly lower during the pandemic compared with either before or after the pandemic (OR, 0.82; 95% CI, 0.75-0.89).

**Table 3.** Association of the COVID-19 Pandemic With Youth Achievement of Healthy Fitness Zone Cardiorespiratory and Musculoskeletal Fitness

Variable	Cardiorespiratory fitness^a^		Musculoskeletal fitness	
	OR (95% CI)	*P* value	OR (95% CI)	*P* value
Female vs male	0.60 (0.59-0.61)	<.001	0.77 (0.76-0.78)	<.001
Grades 7-12 vs 1-6	0.71 (0.68-0.74)	<.001	0.75 (0.72-0.77)	<.001
Title I ineligible vs eligible	2.69 (1.82-3.98)	<.001	1.67 (1.26-2.23)	<.001
Midwest vs Northeast	2.15 (1.33-3.49)	.002	1.14 (0.81-1.61)	.46
South vs Northeast	1.49 (0.94-2.34)	.09	0.83 (0.60-1.15)	.25
West vs Northeast	2.29 (1.35-3.88)	.002	0.89 (0.61-1.29)	.53
During the pandemic vs before or after^b^	0.72 (0.66-0.78)	<.001	0.82 (0.75-0.89)	<.001

Abbreviation: OR, odds ratio.

^a^ Cardiorespiratory fitness decreased during the pandemic for both girls (0.55 mL/kg/min; 95% CI, 0.36-0.74 mL/kg/min) and boys (0.86 mL/kg/min; 95% CI, 0.63-1.10 mL/kg/min), adjusted for the same covariates.

^b^ During the pandemic refers to fall 2020 to fall 2021, before the pandemic refers to fall 2019 to spring 2020, and after the pandemic refers to spring 2022 to spring 2023.

**Figure 2.**
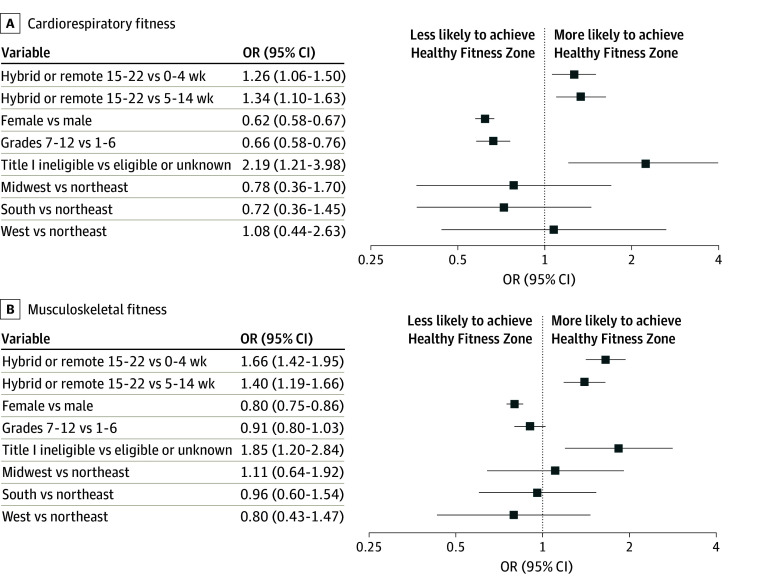
Odds Ratios (ORs) for Healthy Fitness Zone Achievement Based on Learning Modality During the COVID-19 Pandemic Cardiorespiratory and musculoskeletal fitness ORs are adjusted for grade level, sex, Title I eligibility, and region.

In a subset of 116 schools with available learning modality by week, those in remote or hybrid environments for 15 to 22 weeks were significantly more likely to achieve the CRF HFZ (15-22 vs 0-4 weeks, OR, 1.26; 95% CI, 1.06-1.50; 15-22 vs 5-14 weeks, OR, 1.34; 95% CI, 1.10-1.63) compared with those that were hybrid or remote for shorter periods of time (Figure 2). In addition, those in remote or hybrid environments for 15 to 22 weeks were significantly more likely to achieve the HFZ for MSF compared with those that were hybrid or remote for shorter periods of time (15-22 vs 0-4 weeks, OR, 1.66; 95% CI, 1.42-1.95; 15-22 vs 5-14 weeks, OR, 1.40; 95% CI, 1.19-1.66).

## Discussion

This cohort study demonstrated that youth health-related physical fitness, specifically CRF and MSF, decreased significantly during the COVID-19 pandemic. However, although some studies^6,7,8,9^ have speculated that physical activity, and thereby physical fitness, have decreased because of remote learning, these data suggest otherwise. In fact, schools that implemented a remote or hybrid environment for 15 to 22 weeks achieved the HFZ for both CRF and MSF at a greater rate compared with the schools that were remote or hybrid for a shorter period of time. It is possible that other factors, such as social distancing and cancellation of school activities, events, or sports, were greater contributors to decreased activity and fitness than remote or hybrid learning.

To our knowledge, this is the first large study, with data from 264 schools across 21 states and all regions of the US, to examine the association between the COVID-19 pandemic and physical fitness in youths; however, a few substantially smaller studies have evaluated the impact of COVID-19 on anthropometric measurements and physical performance. For example, among 264 children in third through eighth grade in Illinois, Alexander et al^13^ found a significant decline in the PACER and push-up test performance for both male and female students from before to after the COVID-19 pandemic. These findings were coupled with a significant decline in sit-up test performance and an increase in BMI. Similarly, in a sample of slightly younger children (third to fifth graders), Raine et al^14^ found a 53% decline in cardiorespiratory fitness among youths assessed during vs before the onset of pandemic-related shutdowns. Our findings at the school level appear to be consistent with smaller studies using individual-level data, thus, further emphasizing the negative physical health consequences children experienced during the COVID-19 pandemic.

The observed decline in physical fitness coupled with the previously reported^5^ increases in BMI throughout the pandemic are likely the result of decreased physical activity in children. To date, numerous studies^6,7,8,15^ have reported that children and adolescents were significantly less likely to engage in physical activity throughout the pandemic. In fact, in a national survey distributed to PE teachers, 80% reported their students were significantly less active during compared with before the pandemic.^8^ Similar findings were observed when parents reported that children across all age groups had less community-based and peer-related physical activity during the pandemic.^15^ This is likely owing to social distancing requirements and cancellation of group events, specifically sports. It is widely accepted that habitual physical activity drives improvements in physical fitness. As such, in instances where opportunities and access to engage in physical activity were hindered or disrupted, a subsequent decline in physical fitness was likely inevitable.

Another important finding from the present study is that the schools that provided more weeks of in-person learning were significantly less likely to achieve the HFZ for both CRF and MSF. It is possible that students who were required to attend school in-person throughout the COVID-19 pandemic were instructed to social distance, discouraged from interacting with other students, and refrained from excessive movement that might lead to heavier breathing and result in further spread of the virus.^16^ To date, one additional study^17^ has examined the effectiveness of in-person vs remote modality of PE during the COVID-19 pandemic in a smaller sample of schools participating in the present school-based physical activity program. The authors found that the in-person PE was significantly more effective and resulted in more minutes of instruction compared with the remote environment. However, Onadeko et al^17^ only examined PE and did not collect data on other physical activity practices. As such, it is possible that remote or hybrid learning modality allowed youths additional opportunities to increase physical activity throughout the day, thus resulting in higher physical fitness compared with in-person instruction. On the contrary, when students attended PE in person during the pandemic, it is likely their teachers were instructed to keep students social distanced and seated during class, thus eliminating the possibility of being physically active. Finally, Onadeko et al^17^ used survey data collected immediately following the onset of the COVID-19 pandemic. As a result, the total duration of teacher-reported learning modality is unknown, making direct comparisons to the present study difficult.

The strengths of this study include a large sample size with substantial representation both nationally and regionally. The sample of participating schools was racially and ethnically diverse, included both low and high socioeconomic status schools, and represented urban to rural settings of the US. Additionally, other covariates, such as age, sex, and socioeconomic status, were associated with fitness in the well-established directions, thus supporting the accuracy and reliability of our data.

### Limitations

One limitation of the study includes fewer data being collected during the COVID-19 period. This is likely the result of restrictions imposed by the state government and/or school districts. Nonetheless, each school in the sample reported fitness assessment data at least once during the pandemic and an additional time either before or after the pandemic. Furthermore, owing to nonrandom sampling procedures, the study might be subject to selection bias; however, this cohort is composed of schools from all US regions and school settings (eg, urban, rural, town, and suburban), thus making it comparable to the US school population. Another limitation is that the interrater reliability of assessment data collection cannot be measured because the capacity for such control in a very large sample is not possible. However, this evidence-based physical fitness battery is the national assessment for PE that has been administered for more than 4 decades. The methods and protocols of the assessment are widely known and routinely implemented across the nation. The learning modality data provided by the Centers for Disease Control and Prevention were constructed from 4 different sources at the district level and lack reliability and validity testing, which could lead to misclassifying some schools.^18^ Furthermore, we recognize that other, unmeasured, confounding variables could have contributed to the results (eg, sedentary or screen time, mental health concerns, dietary habits, access to physical activity, and so forth).

## Conclusions

In conclusion, to our knowledge, this is the first study to demonstrate a decrease in physical fitness among youths during the COVID-19 pandemic compared with either before or after the pandemic. From fall 2020 to fall 2021 semesters, students in the US were significantly less likely to achieve the HFZ for both CRF and MSF. Prolonged remote or hybrid learning environment was positively associated with HFZ achievement for both components of fitness. As such, the reduction in physical fitness during the pandemic does not appear to be associated with extended remote or hybrid learning. These findings demonstrate the need for strategies aimed at restoring and improving youth fitness through relevant in-school physical activity programs, initiatives, and policies. Should other catastrophic world events (eg, pandemics or wars) occur in the future, health care practitioners, teachers, and parents should have a plan in place aimed at maintaining the physical fitness of youths, thus likely resulting in preserved mental and physical health as well as academic learning.

## Notes

Supplement.
Data Sharing Statement
