# Supplementary material for: Longitudinal Outcomes of the COVID-19 Pandemic on Youth Physical Fitness
Source: JAMA Netw Open. 2025 Jun 4;8(6):e2513721. doi: 10.1001/jamanetworkopen.2025.13721 (PMC12138725; doi:10.1001/jamanetworkopen.2025.13721)
Supplement: Supplement. — Data Sharing Statement [file jamanetwopen-e2513721-s001.pdf]

Data Sharing Statement

Pavlovic. Longitudinal Outcomes of the COVID-19 Pandemic on Youth Physical Fitness. *JAMA Netw Open*. Published June 04, 2025. doi:10.1001/jamanetworkopen.2025.13721

Data
Data available: No

Additional Information
Explanation for why data not available: The Kenneth H. Cooper Institute at Texas Tech University Health Sciences Center (KHCI) and the NFL PLAY 60 FitnessGram Project data are not publicly available. A scientific data request may be submitted to the KHCI's Scientific Review Board Committee for review. The Committee meets regularly to assess the merits of all requests.
